# A rare case of pancreatic glomangiomyoma diagnosed by endoscopic ultrasound-guided fine-needle aspiration

**DOI:** 10.1055/a-2344-7534

**Published:** 2024-07-08

**Authors:** Dafan Chen, Shan Wu, Xinjian Wan

**Affiliations:** 1 Digestive Endoscopic Center, Shanghai Sixth Peopleʼs Hospital Affiliated to Shanghai Jiao Tong University School of Medicine, Shanghai, China

A 67-year-old woman presented with a 3-month history of upper abdominal pain. No positive result was found in tumor markers and antinuclear antibodies. Magnetic resonance imaging (MRI) detected a 15 × 11-mm mass with a T1W low signal and T2W high signal, which was enhanced in the arterial phase and weakened in the delayed phase. Endoscopic ultrasound (EUS) revealed a 14 × 12-mm hypoechoic lesion with a heterogeneous echo and a few stripe hyperechoic tissues. Ultrasound elastography imaging was primarily dominated by yellow-green tones interspersed with small areas of red and blue hues. Contrast-enhanced ultrasound showed heterogeneous enhancement in the arterial phase that receded quickly in the venous phase. The specimens were obtained by EUS-guided fine needle aspiration (FNA) with a 22G puncture needle of wet suction. Pathological examination revealed round tumor cells with SMA(+), Syn(–), Ki67 (3%+), CD34 (vascular+), consistent with the characteristics of a glomangiomyoma ( Fig. 1 ).

**Fig. 1:**
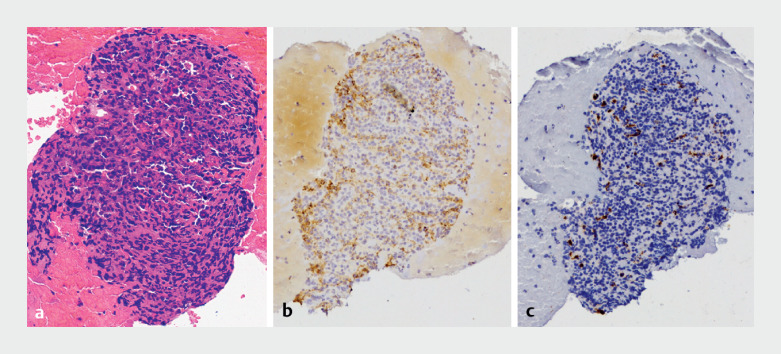
Pathological examination: glomangiomyoma. **a** Hematoxylin-eosin: round tumor cells. **b** SMA(+). **c** CD34(vascular+).

Glomangiomyoma is a rare benign tumor of the glomus body, a specialized form of arteriovenous anastomosis. This case demonstrated the first comprehensive diagnosis procedure for pancreas glomangiomyoma by EUS-FNA. Pancreatic glomangioma was only reported by John R Miliauskas et al. in 2002 1 and Ichiro Tamaki et al. in 2020 2 , which were all diagnosed by surgery. This case showed a well-defined, round-like mass under EUS scanning, and high enhancement under contrast-enhanced ultrasound, similar to neuroendocrine tumors. However, its internal echo was uneven, and under ultrasound elastography imaging, it showed an inhomogeneous soft tissue, whose hardness was lower than most neuroendocrine tumors. Under contrast-enhanced ultrasound, it showed an uneven high enhancement different from the uniform high enhancement of most neuroendocrine tumors. These characteristics might be helpful in differentiating pancreatic hemangiomas from neuroendocrine tumors. EUS-FNA is a reliable and practical technique for diagnosing pancreatic glomus tumors ( Video 1 ).

Clinical manifestation of a rare pancreatic glomangiomyoma.Video 1

Endoscopy_UCTN_Code_CCL_1AF_2AZ_3AB

## Notes

Endoscopy E-Videos https://eref.thieme.de/e-videos
*E-Videos* is an open access online section of the journal *Endoscopy* , reporting on interesting cases and new techniques in gastroenterological endoscopy. All papers include a high-quality video and are published with a Creative Commons CC-BY license. Endoscopy E-Videos qualify for HINARI discounts and waivers and eligibility is automatically checked during the submission process. We grant 100% waivers to articles whose corresponding authors are based in Group A countries and 50% waivers to those who are based in Group B countries as classified by Research4Life (see: https://www.research4life.org/access/eligibility/ ). This section has its own submission website at https://mc.manuscriptcentral.com/e-videos .
